# A Novel Two-Dimensional ZnSiP_2_ Monolayer as an Anode Material for K-Ion Batteries and NO_2_ Gas Sensing

**DOI:** 10.3390/molecules27196726

**Published:** 2022-10-09

**Authors:** Chunying Pu, Zhuo Wang, Xin Tang, Dawei Zhou, Jinbing Cheng

**Affiliations:** 1 Henan International Joint Laboratory of MXene Materials Microstructure, College of Physics and Electronic Engineering, Nanyang Normal University, Nanyang 473061, China; 2 College of mechanical and electrical engineering, Nanyang Normal University, Nanyang 473061, China; 3 College of Material Science and Engineering, Guilin University of Technology, Guilin 541004, China

**Keywords:** two-dimensional ZnSiP_2_, first-principles calculations, K-ion batteries, gas sensing

## Abstract

Using the crystal-structure search technique and first-principles calculation, we report a new two-dimensional semiconductor, ZnSiP_2_, which was found to be stable by phonon, molecular-dynamic, and elastic-moduli simulations. ZnSiP_2_ has an indirect band gap of 1.79 eV and exhibits an anisotropic character mechanically. Here, we investigated the ZnSiP_2_ monolayer as an anode material for K-ion batteries and gas sensing for the adsorption of CO, CO_2_, SO_2_, NO, NO_2_, and NH_3_ gas molecules. Our calculations show that the ZnSiP_2_ monolayer possesses a theoretical capacity of 517 mAh/g for K ions and an ultralow diffusion barrier of 0.12 eV. Importantly, the ZnSiP_2_ monolayer exhibits metallic behavior after the adsorption of the K-atom layer, which provides better conductivity in a period of the battery cycle. In addition, the results show that the ZnSiP_2_ monolayer is highly sensitive and selective to NO_2_ gas molecules.

## 1. Introduction

Two-dimensional semiconductor (2D) materials have potential applications in electronic equipment, catalysis, electrode materials, and gas sensors owing to their significant electrical, physical, and chemical properties [1,2,3,4]. In particular, the large surface areas, excellent mechanical strengths, and strong surface activities of 2D materials provide excellent advantages for the adsorption of certain metal atoms and gas molecules, which make 2D materials suitable as anodes for metal-ion batteries and gas sensors [5,6]. Recently, many novel 2D semiconductors [7,8,9,10,11,12,13,14,15,16,17,18] have attracted much attention due to their high stabilities, good electronic properties, high capacities for metal-ion batteries, and high sensitivities toward certain gases, such as NO_2_, SO_2_, and NH_3_.

As a new family of 2D materials, phosphorus carbides (PCs) with *α* phase and *β* phase are semiconductors that exhibit highly anisotropic electronic characters with high carrier mobilities. More importantly, *α*-PC and *β*-PC, as promising anode materials for Li-, Na-, and K-ion batteries, having high capacities and fast diffusion channels for Li, Na, and K ions [10,11]. It has also been predicted that α-PC, as a promising gas sensor, exhibits superior selectivity and sensitivity for NO_2_ [12]. Buckled-graphene-like PC_6_, as a semiconductor, has been predicted to have ultrahigh carrier mobility and, as an anode for Li-ion batteries, a high capacity of 717 mAh/g and an open-circuit voltage of 0.21 V [13]. Furthermore, typical 2D metal-phosphide δ-InP_3_ exhibits high electron mobility and has been shown to be usable as a N-based gas sensor with high selectivity and sensitivity and good reversibility [16]. In addition, metal oxides, such as two-dimensional WO_3_ and Pd-loaded ZnO monolayers, are important semiconductors applied in gas sensors, with high sensitivities [19,20].

Apart from the excellent performances of binary semiconductors, ternary 2D semiconductor materials have also attracted special interest. Using the epitaxial growth technique, Beniwal and co-workers [21] synthesized a 2D hexagonal graphenic BCN monolayer, which showed semiconductor behavior with a band gap of 1.50 eV, high directional anisotropy, a small Young’s modulus, high flexibility, and suitability as a potential electrode material for Al-based dual-ion batteries [22]. Recently, a new semiconductor BCN structure, by the global-optimization search method, was predicted to have high carrier mobility and excellent optical properties [23]. Using first-principles simulations, two-dimensional BC_2_P and BC_3_P_3_ monolayers were also predicted to present semiconductors with proper band gaps and low barriers for the dissociation of water and hydrogen molecules and thus to show promise for use in renewable energy [24]. Recently, Tang et al. [25] designed a BC_6_P monolayer isostructural and isoelectronic to graphene that has high electron mobility and can be used in K-ion batteries, with a high capacity of 1410 mAh/g.

In recent years, we have noticed that the bulk ternary chalcopyrite-structure compound ZnSiP_2_ is a promising semiconductor that has been synthesized experimentally [26,27] and used for optical, optoelectronic, photovoltaic, and thermoelectric applications [28,29,30,31]. However, its 2D structure is still unclear and has not been studied. In this paper, we predicted a stable structure of the 2D semiconductor ZnSiP_2_ and studied its electronic, mechanical properties as well as its electrode performance for K-ion batteries (KIBs). ZnSiP_2_, as an electrode for K-ion batteries, has a high theoretical storage capacity of 517 mAh/g and a low diffusion energy of 0.12 eV. In addition, its gas-sensing performance was investigated by simulation of the adsorption of CO, CO_2_, SO_2_, NO, NO_2_, and NH_3_ gas molecules on the ZnSiP_2_ monolayer. Our calculation results demonstrate that the strong adsorption ability with respect to K ions and NO_2_ gas molecules on the ZnSiP_2_ monolayer makes it a promising anode for K-ion batteries and gas sensors for NO_2_.

## 2. Results and Discussion

### 2.1. Structure and Stability

**Figure 1:**
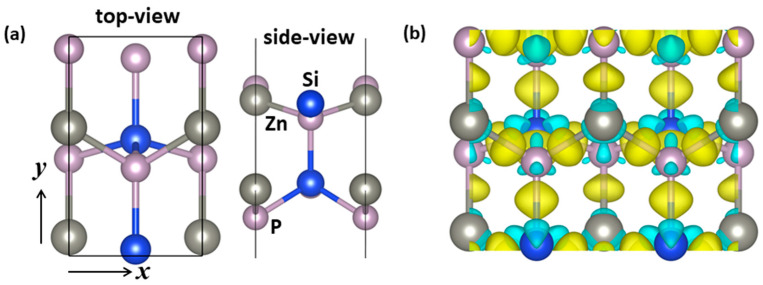
(**a**) The lowest-energy geometry of the ZnSiP_2_ monolayer, with top and side views.(**b**) The charge density difference of the ZnSiP_2_ monolayer. (The gold coloring (i.e., 0.01 e/Å^3^) in the plot indicates an electron-density increase after bonding, and the cyan coloring (i.e., 0.01 e/Å3) indicates a decrease.) Zn atoms are gray, Si atoms blue and P atoms pink.

By using the global-structure search method, we found a new ZnSiP_2_ monolayer with the space group Pmc21 (no. 26) containing two formula units. The structure crystalized in an orthorhombic structure, and the optimized lattice parameters were a = 3.7251 and b = 6.1398 Å. As shown in Figure 1a, a remarkable feature is that the ZnSiP_2_ monolayer is stacked as a bilayer hexagonal lattice, and the two layers are bonded by Si and P, with a distance of 2.286 Å. Each layer was arranged alternately in two kinds of hexagonal rings. One ring was composed of one Si, two Zn, and three P atoms; the other ring was composed of one Zn, two Si, and three P atoms, which gave rise to two types of bonds: Si-P and Zn-P. To understand the chemical-bonding nature, the charge density difference was calculated and shown in Figure 1b, which is defined as the difference between the total electron density of the ZnSiP_2_ monolayer and the charge density of isolated Zn, Si, and P atoms at their specified positions. It can clearly be seen that there is a strong non-polar covalent bond between Si and P [32]. Regarding the Zn-P bonds, the polar covalent bonds between Zn and P atoms were due to the transfer charges shifted toward P atoms.

**Figure 2:**
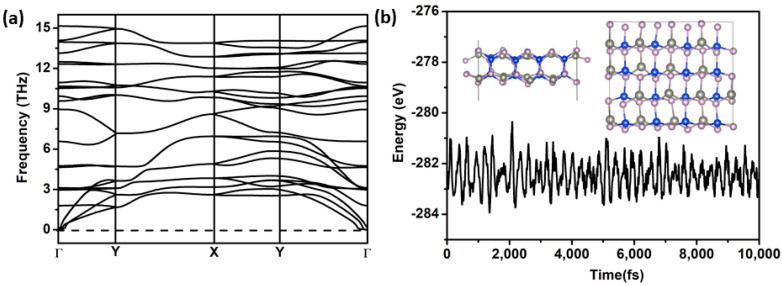
(**a**) The phonon spectra of the ZnSiP_2_ monolayer. (**b**) Vibration of total potential energy of ZnSiP_2_ during the AIMD (400 K). The inset is the final snapshot of ZnSiP_2_ at the end of 10 ps.

The cohesive energy is a key factor in experimental synthesis, which is calculated by $E_{\mathrm{coh}}=(2E_{\mathrm{Si}}+2E_{\mathrm{Zn}}+4E_{P}-E_{{\mathrm{ZnSiP}}_{2}})/8$, where $E_{\mathrm{Si}}$, $E_{\mathrm{Zn}}$, $E_{P}$, and $E_{{\mathrm{ZnSiP}}_{2}}$ represent the energies of one Si, Zn, P, and perfect ZnSiP_2_, respectively. The calculated cohesive energy of the ZnSiP_2_ monolayer was 4.36 eV/atom, which is comparable to those of phophorene (3.30 eV/atom) [33], germanene (3.26 eV/atom), silicene (3.98 eV/atom) [34], and SiP (4.16 eV/atom) [35]. We further calculated the formation energy of the ZnSiP_2_ monolayer related to the SiP_2_ monolayer and Zn metal to investigate its stability, which was calculated by $E_{f}=E_{{\mathrm{ZnSiP}}_{2}}-\mu_{{\mathrm{SiP}}_{2}}-m\mu_{\mathrm{Zn}(\mathrm{bulk})}$, where $\mu_{{\mathrm{SiP}}_{2}}$, $\mu_{\mathrm{Zn}(\mathrm{bulk})}$, and $E_{{\mathrm{ZnSiP}}_{2}}$ are the energies of two-dimensional SiP_2_ [36], one Zn atom in bulk Zn metal, and the perfect ZnSiP_2_ monolayer, respectively, and *m* is the number of Zn atoms. The calculated formation energy is −0.465 eV, the negative value further indicating that the ZnSiP_2_ monolayer may be synthesized. The phonon spectrum was used to check the dynamic stability of the ZnSiP_2_ monolayer. The calculated phonon dispersion curves for the ZnSiP_2_ monolayer are shown in Figure 2a; all frequencies in the Brillouin region were positive, which means that the ZnSiP_2_ monolayer is dynamically stable. Furthermore, thermal stability was checked by AIMD simulation running for 10 ps at 400 K (Figure 2b); the structure remained almost intact at the end of the simulation, revealing that the ZnSiP_2_ monolayer has good thermal stability. According to the above analysis, the predicted 2D ZnSiP_2_ is promising for experimental synthesis.

**Figure 3:**
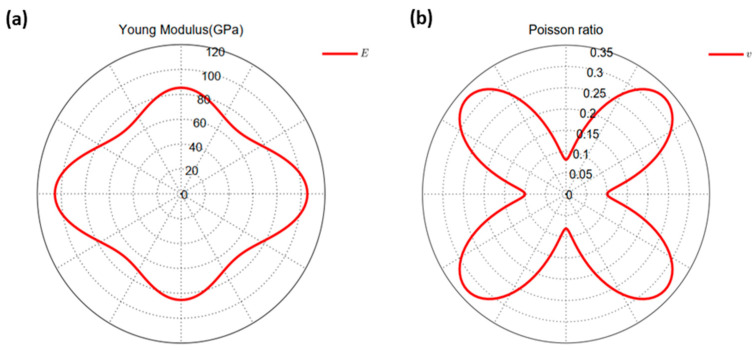
Directional dependences of (**a**) Young’s modulus, E, and (**b**) Poisson’s ratio, υ.

Additionally, we further calculated the four independent elastic constants of the ZnSiP_2_ monolayer, which were $C_{11}=106.2$ Nm^−1^, $C_{22}=86.0$ Nm^−1^, $C_{12}=8.6$ Nm^−1^, and $C_{44}=26.1$ Nm^−1^. According to the obtained elastic constants, the ZnSiP_2_ monolayer satisfied the mechanical stability standard: $C_{11}>0$; $C_{44}>0$; $C_{11}C_{22}>C_{12}^{2}$ [37]. Moreover, the diagrams for the in-plane Young’s modulus and Poisson ratio with polar angle [38] could be obtained and are depicted in Figure 3, showing that the ZnSiP_2_ monolayer is anisotropic. The maximum Young’s modulus (105 N/m) was higher than that reported for phosphorene (92 N/m) [39] and comparable to those of MoS_2_ (129 N/m) [40] and V_2_Te_2_O monolayers (115.3 N/m) [41]. The anisotropy characteristic of mechanical properties also has an important effect on electronic properties.

### 2.2. Electronic and Adsorption Properties

**Figure 4:**
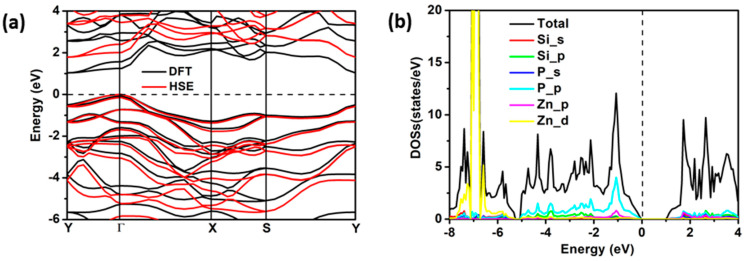
(**a**) The band structure (DFT-PBE and HSE functionals) and (**b**) density of states (PBE functional) for the ZnSiP_2_ monolayer.

The calculated band structure and density of states for the ZnSiP_2_ monolayer are shown in Figure 4a,b. The valence band maximum (VBM) is at point Γ, and the conduction band minimum (CBM) is at point Y. Therefore, as an indirect semiconductor, the band-gap values derived from the PBE and HSE calculations were 1.04 and 1.79 eV, respectively. The band dispersion near the VBM and CBM shows an anisotropic character, which results in the anisotropy of the effective masses. According to the formula $m^{*}=\frac{\hbar^{2}}{\partial E^{2}/\partial k^{2}}$, the obtained electron effective masses near the CBM were 1.364 m_0_ and 0.333 m_0_ along the x-and y-directions, while the hole effective masses near the VBM were 1.019 m_0_ and 0.433 m_0_ along the x- and y-directions, respectively. The density of states in Figure 4b shows that the VBM and CBM are both mainly contributed to by P 2p and Zn 4d orbitals.

**Figure 5:**
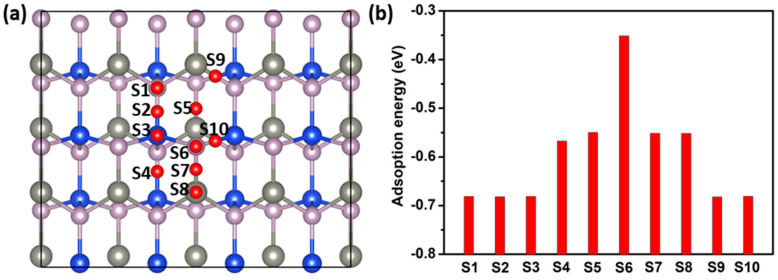
(**a**) S1–S10 are the possible adsorption configurations of K ions on the ZnSiP_2_ monolayer. (**b**) Adsorption energies of K ions at each location.

To further study the performance of the ZnSiP_2_ monolayer as an electrode material, we investigated the adsorption properties of one K atom on its surface using a 3 × 2 × 1 supercell as the substrate. According to the structural symmetry, ten possible K-atom adsorption sites (S1–S10) with adsorption energies based on Equation (1) were considered and calculated, as shown in Figure 5. After geometric-structure optimization, we found some equivalent sites due to the transfer of K atoms from one site to another site. As can be clearly seen in Figure 5b, the equivalent sites were S_1_ = S_2_ = S_3_ = S_9_ = S_10_ and S_5_ = S_7_ = S_8_, so only four sites S_2_, S_4_, S_5_, and S_6_ were left, with adsorption energies of −0.68, −0.57, −0.55, and −0.35 eV, respectively. Thus, the adsorption energy of the K atom at S_2_ site was the lowest, which means that the adsorbed K atoms prefer to stay at the bridge position of Si-P to reduce the Coulomb repulsion between K and Zn. The nearest K-P, K-Zn and K-Si distances are 3.30 Å, 3.92 Å and 3.53 Å, respectively.

**Figure 6:**
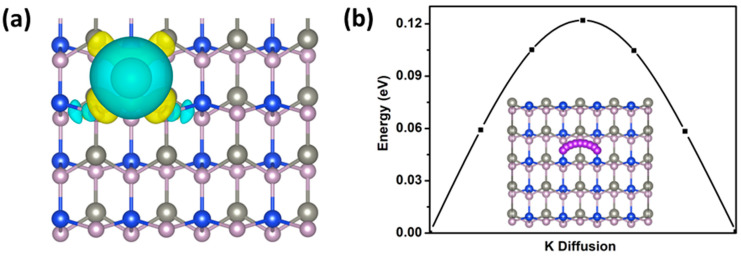
(**a**)The charge density difference with the adsorption of K atom with the isosurface level of 0.01 e/Å^3^. (**b**) Energy profile for the diffusion of K on the surface of ZnSiP_2_ monolayer along the path of the inset. The purple ball represents the K atom.

To assess the adsorption behavior of the K atoms, we calculated the charge-density differences shown in Figure 6a, which is defined by:

$$\Delta \rho =\rho (KZn_{12}Si_{12}P_{24})-\rho (K)-\rho (Zn_{12}Si_{12}P_{24})\;$$

where $\rho (Zn_{12}Si_{12}P_{24})$, $\rho (KZn_{12}Si_{12}P_{24})$, and ρ(K) are the charge densities of the Zn_12_Si_12_P_24_ monolayer with adsorbed K atoms, the substrate Zn_12_Si_12_P_24_, and an isolated K atom, respectively. Obvious charge transfer could be observed, and the K atoms had a net charge of $0.84^{\left|e\right|}$ based on the Bader charge analysis, which implies charge transfer from the K atoms to the adjacent P and Si atoms in the $Zn_{12}Si_{12}P_{24}$ surface.

The diffusion barrier of K ions is a key parameter in estimating the performance of a battery. Next, the diffusion of one K ion on the ZnSiP_2_ surface was investigated. The possible diffusion path (inset of Figure 6b) between the lowest-energy adsorption sites and the calculated results is shown in Figure 6b. The diffusion barrier of the path was 0.12 eV, which is comparable to the result for ReN_2_ (0.127 eV) [42]. Compared with other anode materials, ZnSiP_2_ has a low K-ion diffusion barrier that is smaller than those of BP (0.155 eV) [43], PC_6_ (0.26 eV) [44], and SnC (0.17 eV) [45]. However, this value is larger than those of GeS (0.05 eV) [46], Ti_3_C_2_ (0.103 eV) [47], and C_6_BN (0.087 eV) [48]. The low diffusion barrier can result in ultrafast charging–discharging cycles in K-ion batteries.

### 2.3. Capacity and Open-Circuit Voltage

**Figure 7:**
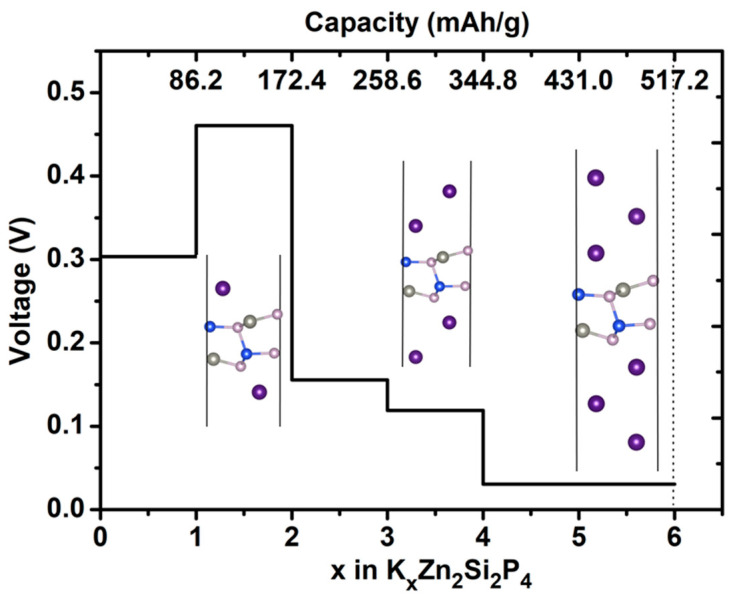
Predicted voltage as a function of capacity and K content (*x*) in K*_x_*Zn_2_Si_2_P_4_.

After studying the adsorption and diffusion behavior of one K atom on the supercell of the ZnSiP_2_ monolayer, we then explored the behavior of K adsorption concentration. Five K concentrations (K*_x_*Zn_2_Si_2_P_4_, *x* = 1–4, 6) were considered, and the average adsorption energies acquired according to Equation (2) were −0.30, −0.46, −0.16, −0.12, and −0.03 eV, respectively. It is to be noted that the K concentration reached *x* = 6, still showing negative adsorption energy, which means that K atoms can be adsorbed on the ZnSiP_2_ monolayer. The three stable adsorption configurations (K_2_Zn_2_Si_2_P_4_, K_4_Zn_2_Si_2_P_4_, and K_6_Zn_2_Si_2_P_4_) are shown in the inset of Figure 7. The first and the second K atom layers are located at S_2_ and S_5_ sites, with both sides of the ZnSiP_2_ monolayer. As for the third K-atom layer, the K atom prefers to stay at the S_2_ site. The stoichiometry K_6_Zn_2_Si_2_P_4_ can provide the maximal storage capacity 517 mAh/g, according to Equation (4), which is higher than other reported values for 2D materials, such as GeS (256 mAh/g) [46], ReN_2_ (250 mAh/g) [42], Ti_3_C_2_ (191 mAh/g) [47], MoS_2_/Ti_2_CS_2_ (141 mAh/g) [49], and MoN_2_ (432 mAh/g) [50], but lower than the capacities for BC_3_ (858 mAh/g) [51], BC_6_P (1410 mAh/g) [25], C_6_BN (533 mAh/g) [48], BP (570 mAh/g) [43], and V_2_S_2_O (883.6Ah/g) [41]. Based on Equation (3), OCVs were obtained and are shown in Figure 7, and the calculated values for different concentrations, KZn_2_Si_2_P_4_, K_2_Zn_2_Si_2_P_4_, K_3_Zn_2_Si_2_P_4_, K_4_Zn_2_Si_2_P_4_, and K_6_Zn_2_Si_2_P_4_, were 0.30, 0.46, 0.16, 0.12, and 0.03 V, respectively. The Bader analysis showed that every K atom transfers 0.58 *e* to ZnSiP_2_ when two K atoms are absorbed on the surface of 2D ZnSiP_2_, while every K atom transfers 0.51 *e* to ZnSiP_2_ when only one K atom is absorbed on the surface of 2D ZnSiP_2_, implying that two K atoms are more easily absorbed on the surface of 2D ZnSiP_2_ than one K atom. So, the OCV increases as *x* increases from 1 to 2, as shown in Figure 7. However, the overall voltage decreases as the capacity increases.

**Figure 8:**
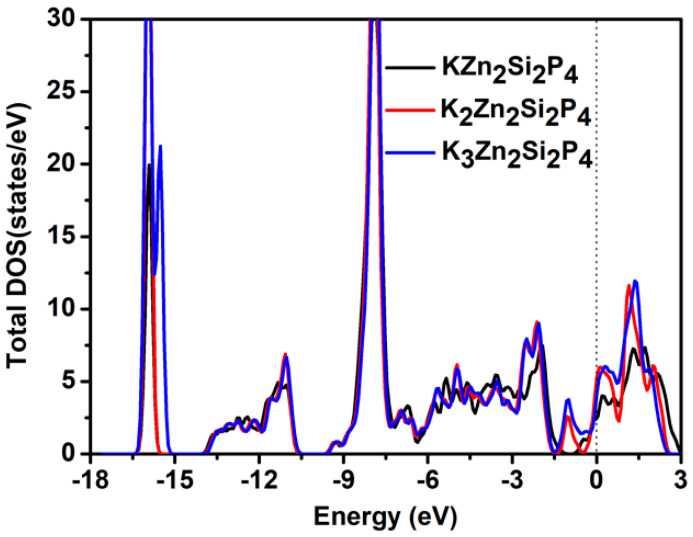
The total densities of states of KZn_2_Si_2_P_4_, K_2_Zn_2_Si_2_P_4_, and K_3_Zn_2_Si_2_P_4_.

Importantly, the density states of the three stable adsorption configurations (KZn_2_Si_2_P_4_, K_2_Zn_2_Si_2_P_4_, and K_3_Zn_2_Si_2_P_4_) were calculated using the PBE functional, and ZnSiP_2_, after the adsorption of K atoms, showed metallic behavior, as shown in Figure 8, which is beneficial for the ZnSiP_2_ monolayer as an electrode material.

### 2.4. Gas-Sensing Properties

**Figure 9:**
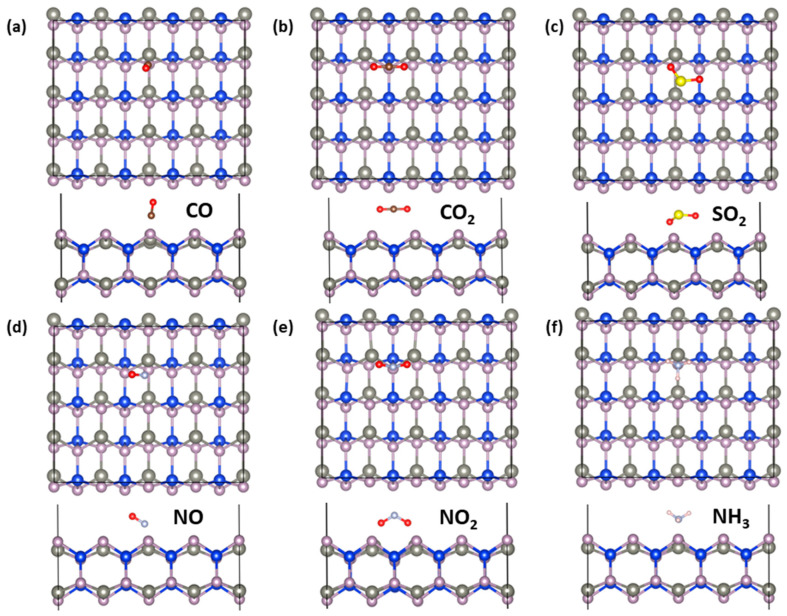
Top and side views of the most stable adsorption of the small gas molecules (**a**) CO, (**b**) CO_2_, (**c**) SO_2_, (**d**) NO, (**e**) NO_2_, and (**f**) NH_3_ on the ZnSiP_2_ monolayer. (The gray, brown, red, yellow, and pink balls represent N, C, O, S, and H atoms, respectively.)

To further study the gas-sensing ability of the ZnSiP_2_ monolayer, we systematically studied the adsorption behavior of gas molecules (CO, CO_2_, SO_2_, NO, NO_2_, and NH_3_) on its surface by first-principles simulations. The most stable configurations of the gas molecules adsorbed on the ZnSiP_2_ monolayer are shown in Figure 9, and the corresponding adsorption energies (*E*_ad_), adsorption distances (*d*_0_), band gaps after molecule adsorption (*E*_g_), and charge transfers (Q) are listed in Table 1. A positive charge for Q means charge transfer from the monolayer to the gas molecules. The equilibrium distance of 1.53 Å between NO_2_ and the monolayer revealed that NO_2_ forms a stable chemical bond. Moreover, the NO_2_ molecules showed high adsorption energies, indicating that ZnSiP_2_ is more sensitive to NO_2_ molecules than the other five molecules. As shown in Table 1, the Bader charge analysis indicated that there were 0.24, 0.12, 0.67, and 0.13 electron transfers between the molecules and the substrates for SO_2_, NO, NO_2_, and NH_3_, which further implies that NO_2_ molecules have strong chemical interactions with the ZnSiP_2_ monolayer.

**Figure 10:**
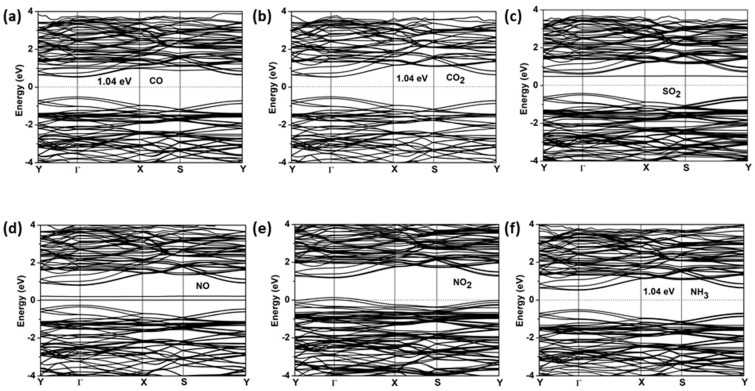
The electronic band structures (PBE functional) for the stable structures of: (**a**) CO, (**b**) CO_2_, (**c**) SO_2_, (**d**) NO, (**e**) NO_2_, and (**f**) NH_3_ adsorbed on the ZnSiP_2_ monolayer.

**Figure 11:**
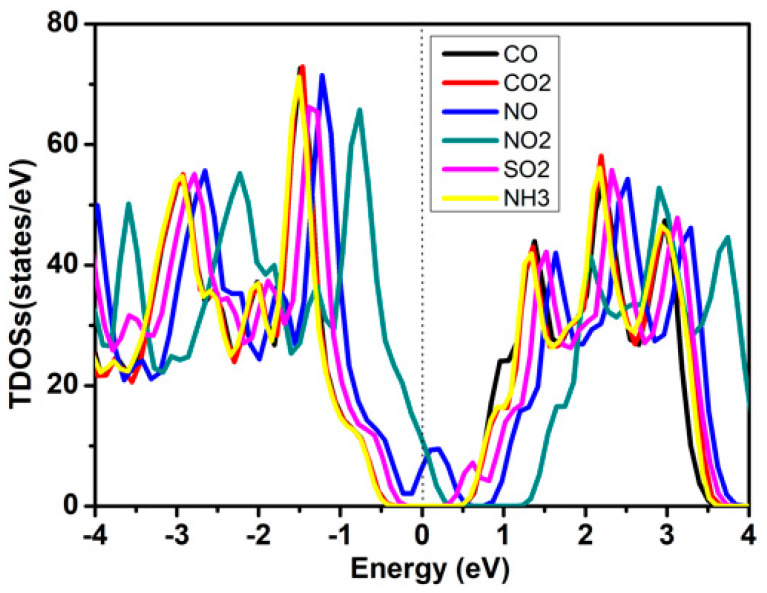
The total densities of states (TDOSs) derived from the PBE functional of the molecules adsorbed on the ZnSiP_2_ monolayer.

The electronic band structures and densities of states for gas-ZnSiP_2_ are shown in Figure 10 and Figure 11, respectively. All the systems, except for NO and NO_2_, that adsorbed the ZnSiP_2_ monolayer became direct band-gap semiconductors, and both VBM and CBM were at the Gamma point. It can be clearly seen from Figure 10 and Figure 11 that the NO and NO_2_ adsorbed on the ZnSiP_2_ monolayer introduced a high density of states at the Fermi surface, which made the ZnSiP_2_ exhibit a metallic character and changed the electronic properties of the ZnSiP_2_ monolayer easily. The adsorption of CO, CO_2_, and NH_3_ had no significant effect on the band structure, and the band gaps did not change much. For SO_2_ adsorption (see Figure 10c), the shallow donor energy levels were introduced into the energy band, resulting in the narrowing of the band gap. Combining all the above results, we can conclude that the ZnSiP_2_ monolayer is promising as a sensor of NO_2_ gas molecules with high selectivity and sensitivity.

## 3. Computational Methods

To find the lowest energy structure of 2D ZnSiP_2_, a swarm-intelligence-based PSO method, implemented in CALYPSO code [52,53], combined with first-principles calculations, was employed, which has been used to successfully predict many 2D systems, such as Cu_2_Si, PC_6_, SnP_3_, and B_2_N_3_ [19,34,54,55]. The structures of 2D ZnSiP_2_ were searched with the simulation cells containing 1–4 formula units. The population size and the number of generations were both set to 30, which have been tested to give convergent results. In the first generation, a population of the structures was generated randomly. In the following generation, 60% of the population was generated from the lowest energy structures in the previous generation and all of the structures were fully relaxed, including the atomic positions and the lattice parameters.

The first-principles calculations based on density functional theory were performed using the projector-augmented wave (PAW) method, as implemented in VASP software [56,57,58]. The exchange correlation potential was described using Perdew–Burke–Ernzerhof (PBE) generalized gradient approximation [59] and corrected by the van der Waals (vdW) interaction in the calculation of the adsorption properties of ZnSiP_2_. The plane-wave energy cut-off and Monkhorst–Pack K-point mesh density were set to 500 eV and 2π × 0.03 Å^−1^, respectively. All geometries were optimized and relaxed until a total energy change smaller than 10^−6^ eV and a force tolerance acting on each atom less than 0.001 eVÅ^−1^ was achieved. In order to make the band-gap calculation more accurate for semiconductors, the HSE06 functional was employed [60]. A vacuum thickness of 25 Å was used to avoid the interlayer interactions. The nudged elastic band (NEB) method was used to obtain the K-ion diffusion energy barrier. To assess the dynamic stability, phonon spectra were calculated using the PHONOPY code [61]. In addition, ab initio molecular dynamics (AIMD) were explored with the NVT ensemble to examine the thermal stability.

In order to study the interactions between metals (gas molecules) and substrates, adsorption energies and adsorption distances were systematically calculated, according to the following equation:

(1) $$E_{\mathrm{ad}}=\frac{\left(E_{\mathrm{total}}-nE_{\mathrm{metal}(\mathrm{gas})}-E_{{\mathrm{ZnSiP}}_{2}}\right)}{n}$$

where $E_{\mathrm{total}}$, $E_{{\mathrm{ZnSiP}}_{2}}$, and $E_{\mathrm{metal}(\mathrm{gas})}$ represent the total energy of the metal (gas molecules) adsorbed on the ZnSiP_2_ monolayer, the perfect ZnSiP_2_ monolayer, and the metal in the bulk metal or gas molecules, respectively, and *n* is the number of adsorbed metal atoms.

The adsorption stability of the K-ion layer on the ZnSiP_2_ monolayer is estimated by average adsorption energy, which is calculated using the following formula:

(2) $$E_{\mathrm{av}}=\frac{E_{n\mathrm{total}}-E_{(n-1)\mathrm{total}}-mE_{K}}{m}$$

where $E_{n\mathrm{total}}$ and $E_{(n-1)\mathrm{total}}$ refer to the total energies of the ZnSiP_2_ monolayer with *n* and (*n*−1) layers and *m* is the number of K atoms in every layer.

For a given concentration *x* of K*_x_*Zn_2_Si_2_P_4_, the open-circuit voltage (OCV) can be obtained with the following equation:

(3) $$V=\frac{E(x_{2})-E(x_{1})-(x_{2}-x_{1})E_{K}}{e(x_{2}-x_{1})}$$

where $E(x_{2})$ and $E(x_{1})$ are the total energies of K_x_Zn_2_Si_2_P_4_ at two adjacent K-ion concentrations $x_{2}$ and $x_{1}$, *e* is the element charge, and $E_{K}$ is the energy of one K atom in the bulk K metal.

The theoretical capacity can be evaluated from:

(4) $$C_{M}=\frac{cF}{M}$$

where *c* is the number of adsorbed K atoms per ZnSiP_2_ unit, F is the Faraday constant (26,801 mAhmol^−1^), and *M* is the molar weight of ZnSiP_2_ in gmol^−1^.

## 4. Conclusions

In summary, we predicted the ZnSiP_2_ monolayer as a new 2D semiconductor material which can be used as an anode material for K-ion batteries and NO_2_ gas sensors by the global-optimization algorithm combined with first-principles calculation. Phonon simulation, molecular dynamics, and elastic-constant calculations confirmed its stability. The calculated electronic structure and mechanical properties indicate that ZnSiP_2_ has an indirect band gap of 1.79 eV and exhibits anisotropic mechanical characteristics. Furthermore, we investigated 2D ZnSiP_2_ as an anode for KIBs. The ZnSiP_2_ monolayer has a theoretical capacity of 517 mAh/g for K-ions and a low diffusion barrier of 0.12 eV. In addition, we also investigated the gas-sensing properties of the ZnSiP_2_ monolayer with six gas molecules (CO, CO_2_, SO_2_, NO, NO_2_, and NH_3_). The results show that the ZnSiP_2_ monolayer is a promising gas sensor for NO_2_ with high sensitivity and selectivity.

## Figures and Tables

**Table 1:** The adsorption energy, equilibrium distance, energy band gap, and charge transfer for different gas molecules adsorbed on the ZnSiP_2_ monolayer.

Molecule	CO	CO_2_	SO_2_	NO	NO_2_	NH_3_
*E*_ad_ (eV)	−0.74	−0.55	−1.09	−0.75	−1.30	−1.14
*d*_0_ (Å)	1.54	2.29	1.73	1.68	1.53	1.53
*E*_g_ (eV)	1.04	1.04	0.9	metal	metal	1.04
Q (e)	0	0	−0.24	0.12	0.67	−0.13

## Data Availability

Data available on request from the authors.

## Sample Availability

Samples of the compounds are available on request from the authors.
